# Comparing the efficacy of efavirenz and boosted lopinavir using viremia copy-years

**DOI:** 10.7448/IAS.17.1.18617

**Published:** 2014-05-06

**Authors:** Viviane D Lima, Juan Sierra-Madero, Zunyou Wu, Joel Singer, Evan Wood, Mark W Hull, Paul Richard Harrigan, Julio SG Montaner

**Affiliations:** 1 British Columbia Centre for Excellence in HIV/AIDS, St. Paul's Hospital, Vancouver, BC, Canada; 2 Division of AIDS, Faculty of Medicine, University of British Columbia, Vancouver, BC, Canada; 3 Instituto Nacional de Ciencias Médicas y Nutrición Salvador Zubirán, Mexico City, Mexico; 4 Chinese Center for Disease Control and Prevention, Beijing, China; 5 School of Population and Public Health, University of British Columbia, Vancouver, BC, Canada; 6 CIHR Canadian HIV Trials Network, Vancouver, BC, Canada

**Keywords:** HIV-1 plasma viral load, antiretroviral therapy, clinical trial, area under the curve, cumulative viremia, efficacy, efavirenz, boosted lopinavir

## Abstract

**Introduction:**

HIV-1 plasma viral load during treatment can be highly variable. Thus, there is the need to find a measure of cumulative viremia that can be used to assess both the short- and long-term efficacy of highly active antiretroviral therapy (HAART). Here, we validate a measure of cumulative viremia to evaluate HAART efficacy.

**Methods:**

We accessed HAART efficacy using data from a randomized clinical trial conducted in Mexico. We compared the proportion of individuals achieving a viral load <50 and <400 copies/mL at week 48, against the cumulative plasma viral load, estimated as the area under the plasma viral load curve (AUVLC). High AUVLC indicates high cumulative viremia.

**Results and discussion:**

There was a strong and significant association between the proportion of individuals achieving a viral load <50 and <400 copies/mL at week 48, with individuals suppressed having significant lower cumulative viremia. The median area was 7513 (25th–75th percentile [Q1–Q3] 6634−8180) if viral load <50 copies/mL and 7679 (Q1–Q3 6899−9373) if viral load ≥50 copies/mL (*p*-value 0.0284). When the analysis was stratified by study arm, individuals on efavirenz had lower cumulative viremia than those on boosted lopinavir.

**Conclusions:**

Our findings suggest that cumulative viremia should be explored further as a tool to simultaneously evaluate the individual and public health efficacy of HAART. This is particularly relevant to the implementation and evaluation of the Treatment 2.0 strategy recently proposed by UNAIDS and the WHO, as a means to maximize the individual and public health benefit of HAART.

## Introduction

The goal of highly active antiretroviral therapy (HAART) is to increase disease-free survival through sustained full suppression of viral replication (i.e. HIV-1-RNA plasma viral load <50 copies/mL) [1]. The relative efficacy of candidate HAART regimens has typically been evaluated in clinical trials and observational studies based on the rate of suppression of plasma HIV-1 RNA levels (hereafter referred to viral load) at a pre-specified time point during follow-up, most frequently at 48 weeks. However, viral load can vary during a patient's treatment history. This can be due to a number of factors, including incomplete adherence, inter-current illnesses, immunizations, or even technical issues related to the assay used [2–5]. It is, therefore, important that new validated endpoints be explored to access HAART's efficacy that incorporate the entire patient viremia history. The objective of this study is to evaluate the use of cumulative viral load (hereafter referred to as cumulative viremia) as a novel candidate tool to evaluate HAART efficacy at 48 weeks of follow-up. We hypothesize that patients with high cumulative viremia will also have unsuppressed viral load at 48 weeks.

## Methods

### Study population

The randomized clinical trial NCT00162643 has been described in detail elsewhere [6]. Briefly, this was a prospective open label randomized trial conducted in 10 clinical sites from five different states in Mexico. Eligible participants were ≥18 years old, naïve to HAART and with a CD4 cell count <200 cells/mm^3^ and a viral load ≥1000 copies/mL. Recruitment was carried out from 1 January 2005 to 31 January 2007 and clinical evaluations were conducted at baseline, and at weeks 8, 24, 32 and 48. Patients started HAART containing two nucleoside reverse transcriptase inhibitors selected by the treating physician, in combination with either efavirenz (a non-nucleoside reverse transcriptase inhibitor – NNRTI), or lopinavir\r (a boosted protease inhibitor – PI\r). Viral load was monitored at each visit using the Roche Amplicor HIV-1 Monitor ultrasensitive assay (Roche Diagnostic Systems, Branchburg, NJ, USA – limits of quantification ranging from 50 to 75,000 copies/mL). For our calculations, values <50 copies/mL received the value 49 copies/mL and values >75,000 copies/mL received the value 75,010 copies/mL. This re-coding of viral load values is consistent with manuscripts published by our group throughout the years.

### Outcome variables and statistical analyses

The measure of cumulative exposure to viral replication or cumulative viremia was estimated as the area under the viral load curve (AUVLC). We compared the AUVLC against the traditional endpoint of viral load (<50 and <400 copies/mL) at week 48 using data from the randomized clinical trial. The primary endpoint of the trial was the proportion of patients achieving a viral load <50 copies/mL at week 48, and for the secondary endpoint, viral suppression was defined as <400 copies/mL at week 48. We used an intent-to-treat approach in these analyses. Viral load measurements were obtained at baseline and at weeks 8, 24, 32 and 48.

**Figure 1:**
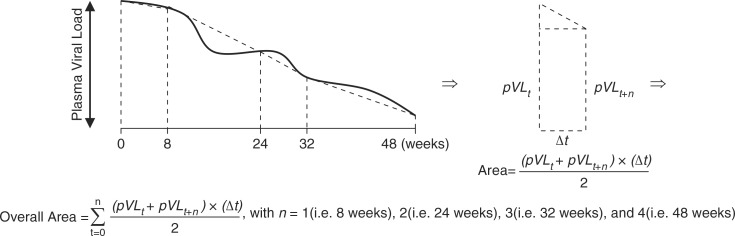
Trapezoidal rule to obtain the area under the plasma viral load of each patient enrolled in the randomized clinical trial, with plasma viral load measured at baseline and at weeks 8, 24, 32 and 48.

We also measured the AUVLC over 48 weeks, as a measure of a patient's cumulative exposure to HIV while on HAART. The AUVLC was calculated using the trapezoidal rule (Figure 1), whereby larger areas are indicative of higher cumulative viremia. We calculated the AUVLC using viral load measurements in copies/mL and log_10_ copies/mL, and therefore, the AUVLC was expressed, respectively, as copy-years/mL and log_10_ copy-years/mL. The trapezoidal rule derives from calculus in which the area under a curve between two distinct points can be found by calculating a definite integral between these points. This methodology is extensively used in toxicology, biopharmaceutics, pharmacokinetics and clinical epidemiology. We have previously used this methodology to summarize the longitudinal CD4 cell count measurements and assess its relationship with regimen adherence [2].

In all bi-variable statistical analyses, categorical variables were compared using the Fisher's exact test, and continuous variables were compared using the Wilcoxon rank sum test. We also estimated the correlation of viral loads over time using the Spearman correlation coefficient. All analyses were performed using SAS software version 9.3 (SAS, Cary, NC).

## Results

Overall, 189 patients were included in this study. Among the 189 patents, 161 (85%) were male, 95 (50%) received efavirenz-based HAART, and 165 (87%) had a baseline viral load >75,000 copies/mL. The median age was 35 years (25th–75th percentile [Q1–Q3]: 29–42 years) and the median CD4 cell count was 56 cells/mm^3^ (Q1–Q3: 25–117 cells/mm^3^). In this setting, the median number of viral loads per patient that contributed to the AUVLC was 5 (Q1–Q3: 4–5 measurements).

The plasma viral loads during weeks 8, 24, 32 and 48 were highly correlated. Of the 188, a total of 127 (67%) patients had measurements at all time-points. The pattern of missing viral load was as follows: 18 (10%) patients had viral load missing at all visits, and the remaining patients (*N*=44; 23%) had viral loads missing at random. For the efavirenz-based HAART arm, the median viral loads (in log_10_ copies/mL) were 4.88 (Q1–Q3: 4.88–4.88) at baseline, 1.97 (Q1–Q3: 1.69–2.56) at week 8, and 1.69 (Q1–Q3: 1.69–1.69) for weeks 24, 32 and 48. For the lopinavir\r-based HAART, the median viral loads (in log_10_ copies/mL) were 4.88 (Q1–Q3: 4.88–4.88) at baseline, 2.64 (Q1–Q3: 2.20–3.21) at week 8, and 1.69 (Q1–Q3: 1.69–1.69) for weeks 24, 32 and 48.

A total of 117 (62%) patients achieved suppressed viral load based on the primary endpoint, while 13 (7%) patients were suppressed only on the basis of the viral load <400 copies/mL criterion. The median AUVLC was 7513 (Q1–Q3: 6189–8165) copy-years/mL for those suppressed (i.e.<50 copies/mL) and 7660 (Q1–Q3: 6874–9353) copy-years/mL for those detectable (i.e. ≥50 copies/mL; *p*-value 0.0284). In terms of log_10_ AUVLC, the results were 1.62 (Q1–Q3: 1.48–1.75 log_10_ copy-years/mL) and 1.71 (Q1–Q3: 1.32–1.93 log_10_ copy-years/mL), respectively. Similar results were obtained when viral load <400 copies/mL was used as an endpoint.

A total of 67 (71%) patients on the efavirenz-based arm achieved a viral load <50 copies/mL, while 50 (53%) patients achieved viral suppression on the lopinavir\r-based arm (*p*-value 0.0167) (Table 1). When we compared the AUVLC between the two treatment arms, patients on efavirenz-based HAART had lower AUVLC over 48 weeks, using AUVLC calculated in log_10_ copy-years/mL (1.56 versus 1.75 log_10_ copy-years/mL; *p*-value 0.0002) (Table 1). In a second analysis, given that the viral load distribution of patients at the start of HAART is highly heterogeneous, we excluded viral loads prior to six months. The results also showed that patients on efavirenz-based HAART had lower AUVLC over 48 weeks, using AUVLC calculated in log_10_ copy-years/mL (0.26 versus 0.28 log_10_ copy-years/mL; *p*-value 0.0132) (Table 1).

**Table 1:** Comparison of virologic outcomes by different third drugs in a HAART regimen after 48 weeks in the randomized clinical trial

Third antiretroviral in the HAART combination	Trial outcome at week 48			
	*Primary n (%)*		*Secondary n (%)*	
	
	<50 copies/mL	>50 copies/mL	<400 copies/mL	>400 copies/mL
	
Efavirenz	67 (71%)	28 (29%)	69 (73%)	26 (27%)
Lopinavir\r	50 (53%)	44 (47%)	61 (65%)	33 (35%)
	*p*-value: 0.0167		*p*-value: 0.2746	
	*Plasma viral load AUC*			
	
	Viral loads since baseline		Viral loads after six months	
	
	copy-years/mL	log10 copy-years/mL	copy-years/mL	log10 copy-years/mL
	
Efavirenz	7423.5 (6649.91–8081.07)	1.56 (1.46–1.68)	7.65 (7.52–9.26)	0.26 (0.26–0.30)
Lopinavir\r	7859.98 (6811.39–9051.12)	1.75 (1.51–1.92)	8.59 (7.52–13.02)	0.28 (0.26–0.32)
	*p*-value: 0.0711	*p*-value: 0.0002	*p*-value: 0.2110	*p*-value:0.0132

Values are the median and the values in parenthesis are the 25th and 75th percentiles.

## Discussion

Our results demonstrate that cumulative viremia, estimated as the AUVLC in log_10_ copy-years/mL, is comparable to the proportion of patients achieving a viral load <50 at week 48 within a comparative trial of NNRTI versus PI/r-based HAART. This measure of cumulative viremia is able to differentiate between a person who is not suppressed at week 48 because a unique blip, with a low overall AUVLC, from a person who is truly not suppressed, with a high overall AUVLC. This measure, therefore, tells us a lot more than just that one value at week 48.

Our results extend the value of the cumulative viremia, originally proposed by Cole and colleagues under the name of copy-years viremia as an independent prognostic marker for the risk of progression to AIDS or death among untreated HIV infected men in the MACS cohort [7,8]. Taken together, these results suggest that cumulative viremia can be used as an independent prognostic marker for the risk of progression to AIDS or death in natural history studies and as a HAART efficacy endpoint in randomized clinical trials. Further analyses are needed in larger clinical trials and in trials evaluating the efficacy of other antiretrovirals with potentially very distinct patient responses (e.g. integrase inhibitors) to conclusively establish the use of copy-years viremia as a valid and robust trial endpoint.

As in Mugavero *et al*. [8], we suggest that cross-sectional viral loads should be used in conjunction with cumulative viremia. Both measures are important to understand a patient's response to HAART. Since 2000, several studies also demonstrated the link between viral load and the likelihood of HIV transmission [9]. Thus, the main advantage of using cumulative viremia is that it captures the overall viral load trajectory of a patient. Consider the following four viral load scenarios for baseline, weeks 8–48 and cumulative viremia, respectively: (1) 20,000, 1310, 49, 49 and 49 copies/mL and 2139.09 copy-years/mL; (2) 31,600, 614, 1170, 49 and 49 copies/mL and 3249.48 copy-years/mL; (3) 50,000, 396, 49, 49 and 202 copies/mL and 5392.75 copy-years/mL; and (4) 75,049, 815, 49, 49 and 75,049 copies/mL and 6063.71 copy-years/mL. Take scenarios 3 and 4 in which there was no suppression at week 48 and compare all viral loads and AUC. If we only look whether there was suppression or not at week 48, we would not see that scenario 4 had a higher contribution to HIV transmission and a higher chance of future disease progression (higher AUC) than scenario 3. Take the scenarios with suppression at week 48 and notice the different trajectories of the viral loads. Again, if we only take the last viral load, we would miss the heterogeneity in viral load distribution in the data.

Based on these results, cumulative viremia deserves further evaluation particularly as a means to evaluate HAART roll-out strategies in environments where real-time viral load are available, or where there may be incomplete adherence. In these instances, the use of a measure of cumulative viremia may be highly advantageous to compare the relative effectiveness of different HAART regimens with regards to their long-term efficacy in suppressing viremia, resilience to acquired drug resistance and preventing mortality. It is important to remember that to calculate cumulative viremia, it requires repeated viral load testing. This limitation should not pose a problem in resource-rich settings with readily available viral load testing. In resource-limited settings, point of care viral load testing can be adopted to calculate this measure.

As stated in our objective, at week 48, we compared the AUVLC against the primary and secondary endpoints of the trial. In these analyses, we observed that the AUVLC not using log_10_ is susceptible to extreme observations as per its interquartile range (IQR) for both study arms, i.e. the IQR for efavirenz-based HAART was 1431 (approximately 3.2 log_10_) and for lopinavir\r-based HAART it was 2240 (approximately 3.4 log_10_) which were three times larger than the IQR for both arms using the AUVLC using log_10_. This phenomenon requires more investigation in a setting with more viral load measurements available. Second, in our analysis restricting, the viral load measurements to after the first six months were consistent with the overall analyses. However, since this analysis only included viral load measurements at weeks 24, 32 and 48, these results should be interpreted with caution. In a sensitivity analysis, we excluded the viral load measurements drawn at week 48 to calculate the area under the curve, and we compared the cumulative viremia results with the primary and secondary endpoints to assess the robustness of this new endpoint. The results from this analysis were also consistent with the main analysis in this study. The last sensitivity analysis only included patients with viral loads measured at all visits. The results from this last analysis were also consistent with the main results in this study.

## Conclusions

In summary, our results indicate that cumulative viremia potentially represents an adequate endpoint for evaluating HAART efficacy in clinical trials. This measure of cumulative viremia captures the full viral load trajectory over time; therefore, it may be ideally suited to evaluate the impact of HAART on HIV transmission. As such, cumulative viremia should be explored further as a tool to simultaneously evaluate the individual and public health efficacy of antiretroviral therapy.
